# Colorimetric Humidity Sensors Based on Electrospun Polyamide/CoCl_2_ Nanofibrous Membranes

**DOI:** 10.1186/s11671-017-2139-0

**Published:** 2017-05-19

**Authors:** Ming-Hao You, Xu Yan, Jun Zhang, Xiao-Xiong Wang, Xiao-Xiao He, Miao Yu, Xin Ning, Yun-Ze Long

**Affiliations:** 1 Collaborative Innovation Center for Nanomaterials & Devices, College of Physics, Qingdao University, Qingdao, 266071 China; 2 Industrial Research Institute of Nonwovens & Technical Textiles, College of Textiles & Clothing, Qingdao University, Qingdao, 266071 China; 3 Department of Mechanical Engineering, Columbia University, New York, NY 10027 USA

**Keywords:** Colorimetric humidity sensor, Electrospinning, PA66/CoCl_2_ nanofibrous membranes

## Abstract

Humidity indicators based on composite polyamide 66/cobalt chloride (PA66/CoCl_2_) nanofibrous membranes (NFMs) were successfully fabricated by electrospinning. A series of NFMs with various weight percentage of CoCl_2_ to PA66 were prepared, and their humidity sensitivity based on color changing and quartz crystal microbalance (QCM) were studied. Due to the color change property of cobalt chloride, the as-spun composite NFMs show obviously macroscopic color change from blue to pink as relative humidity (RH) increasing from 12.4 to 97.2%. Moreover, the QCM detection showed a linear dependence on the RH changing and exhibited short response/recovery time (less than 65.4 s/11 s), small hysteresis (less than 11%), good reproducibility, and stability. Owing to the above double sensitive mechanism on RH, the PA66/CoCl_2_ composite NFM may show great potential applications from meticulous to coarse.

**Electronic supplementary material:**

The online version of this article (doi:10.1186/s11671-017-2139-0) contains supplementary material, which is available to authorized users.

## Background

Relative humidity (RH) sensor is mainly used for monitoring atmospheric humidity environment and shows important applications in warehousing [1], environmental monitoring [2], instruments and meters, and meteorology [3]. Until now, various types of humidity sensors have been reported, such as resistance type [4], capacitor type [5], field-effect-transistor type [6], optical type [7, 8], and so on. Among these types of sensors, the optical type has attracted a lot of interests since it is supplying a change in optical properties, easily detectable with the naked eye (visual) which is suitable for applications in daily life [8–10].

It is now believed that RH sensors based on the electrospun nanofibrous membranes (NFMs) show improved sensor sensitivity due to their large surface area to volume ratio [11–13], providing an increased number of sites for analyte interaction or signal transduction [10, 14–17]. For example, electrospun polyamide 6 nano-fiber/net modified by polyethyleneimine was investigated as RH indicator detected by quartz crystal microbalance (QCM) [14]. It shows high sensitivity and the response and recover times are 120 and 50 s, respectively, with RH changing from 2 to 35% [14]. Moreover, ceramic LiCl-doped ZnO electrospun fibers were also fabricated as humidity sensor, with response time and recovery time about 3 and 6 s [15].

While the sensitivity of the above humidity sensors based on electrospun fibers were detected by precise instrument, [14, 15] it may do not work in practical application limits to conditions. Optical humidity sensors will be a possible solution. Since RH do not have measurable intrinsic optical properties, some intermediate agent will be introduced to show a change in optical property. At present, several intermediate agents have been adapted to realize colorimetric indicator for humidity including photonic crystal [8, 9, 18], polymer electrolyte thin films [19], doped cholesteric liquid crystal [20], and crystalline covalent organic framework nanofibers [21]. In addition, cobalt chloride has been applied as colorimetric RH indicator for its color changes when contaminated by water [7]. Typical colorimetric RH indicator such as silica gel self indicator and CoCl_2_-based optical humidity sensor [7, 22] have been presented. However, colorimetric humidity sensor based on electrospun fibers was rarely investigated.

In this work, we fabricated PA66/cobalt chloride NFMs by electrospinning and investigated its humidity sensing properties by extensive color change and both fine quartz crystal microbalance (QCM). The results showed that the electrospun PA66/cobalt chloride NFM sensors exhibiting high humidity sensitivity with obviously color change, rapid response/recovery performance, small hysteresis, excellent reproducibility, and good stability.

## Methods

### Preparation of Polymer/Cobalt Chloride Solutions

The solutes were CoCl_2_·6H_2_O (Sinopharm Chemical Reagent Co., Ltd) and PA66 (Tianjin Heowns Biochem LLC., China). As a comparison, the concentrations of CoCl_2_·6H_2_O in these solutes were 0 (pure PA66), 10, 30, and 50 wt%, respectively. Dissolved these solutes in formic acid at 12 wt% and stirred thoroughly for 6 h at room temperature, then the uniform precursor solutions were obtained.

### Fabrication of Colorimetric Nanofiber Membranes for Humidity Detection

**Fig. 1:**
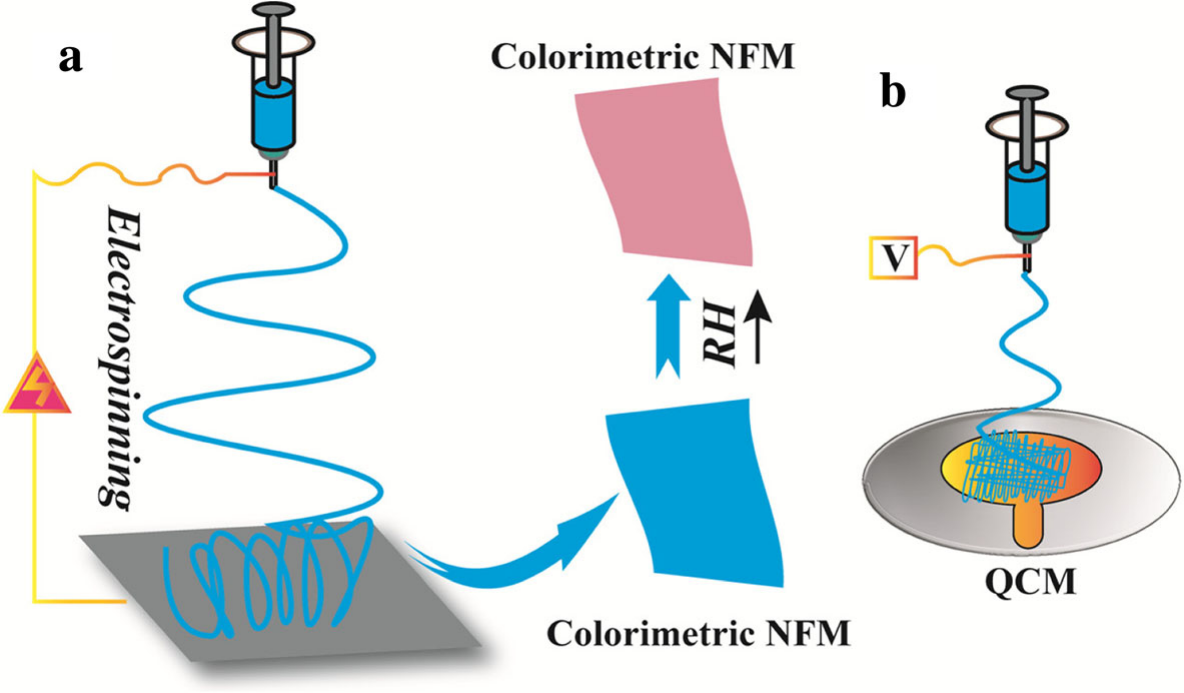
Schematic illustrations of the electrospinning process to fabricate hybrid PA66/cobalt chloride NFM. **a** Fabrications of colorimetric NFM for humidity detection. **b** Illustration of depositing NFMs on the electrode surface of the QCM

The process of fabricating fiber membranes by electrospinning is depicted in Fig. 1a. Firstly, the precursor solution was loaded into a syringe and regulated the flow rate of the solution at 10 μL min^−1^ by a syringe pump (LSP01-1A, Baoding Longer Precision Pump Co., Ltd., China). The applied voltage was kept at 18 kV, and the distance between the needle and collector was about 15 cm. The ambient humidity was controlled at 50–60% RH, and the temperature was between 20 and 25 °C. Electrospinning for about 30 min, the as-spun composite NFMs could be obtained.

### Fabrication of Sensing Membranes on QCM

To further investigate the humidity sensitivity of the as-spun NFM, we also selected QCM for precision measurement. The process of fabricating sensing NFMs on the QCM (CHI400C, Shanghai Huachen Instruments Co., Ltd., China) chip is showed in Fig. 1b. Under the above electrospinning conditions, PA66/cobalt chloride NFMs were deposited onto the surface of the QCM chip, and then the humidity sensitivity can be measured.

### Characterization

The photographs of the colorimetric NFMs were recorded by a digital camera (DSC-TX9C 50i). Absorption spectra were recorded by a UV-vis spectrometer (U-4100, Hitachi) under absorption mode. The morphologies and structures of the NFMs were characterized by a scanning electron microscopy (SEM, EVO MA 10/LS 10, CARL ZEISS Co., Ltd., Germany) and a transmission electron microscope (TEM, JEM-2100PLUS, JEOL Ltd., Japan). Fourier transform infrared (FT-IR) spectra were recorded on a Thermo Scientific Nicolet In10 spectrometer. Means for humidity control are saturated salt solution humidity bottles, which have a stable humidity environments of 12.4% RH (Saturated LiCl solution, in 20 °C), 33.6% RH (Saturated MgCl_2_ solution, in 20 °C), 55.2% RH (Saturated Na_2_Cr_2_O_7_·2H_2_O solution, in 20 °C), 75.5% RH (Saturated NaCl solution, in 20 °C), and 97.2% RH (Saturated K_2_SO_4_ solution, in 20 °C), respectively.

## Results and Discussion

### Colorimetric Property of the As-Spun NFM upon Exposure to Humidity

**Fig. 2:**
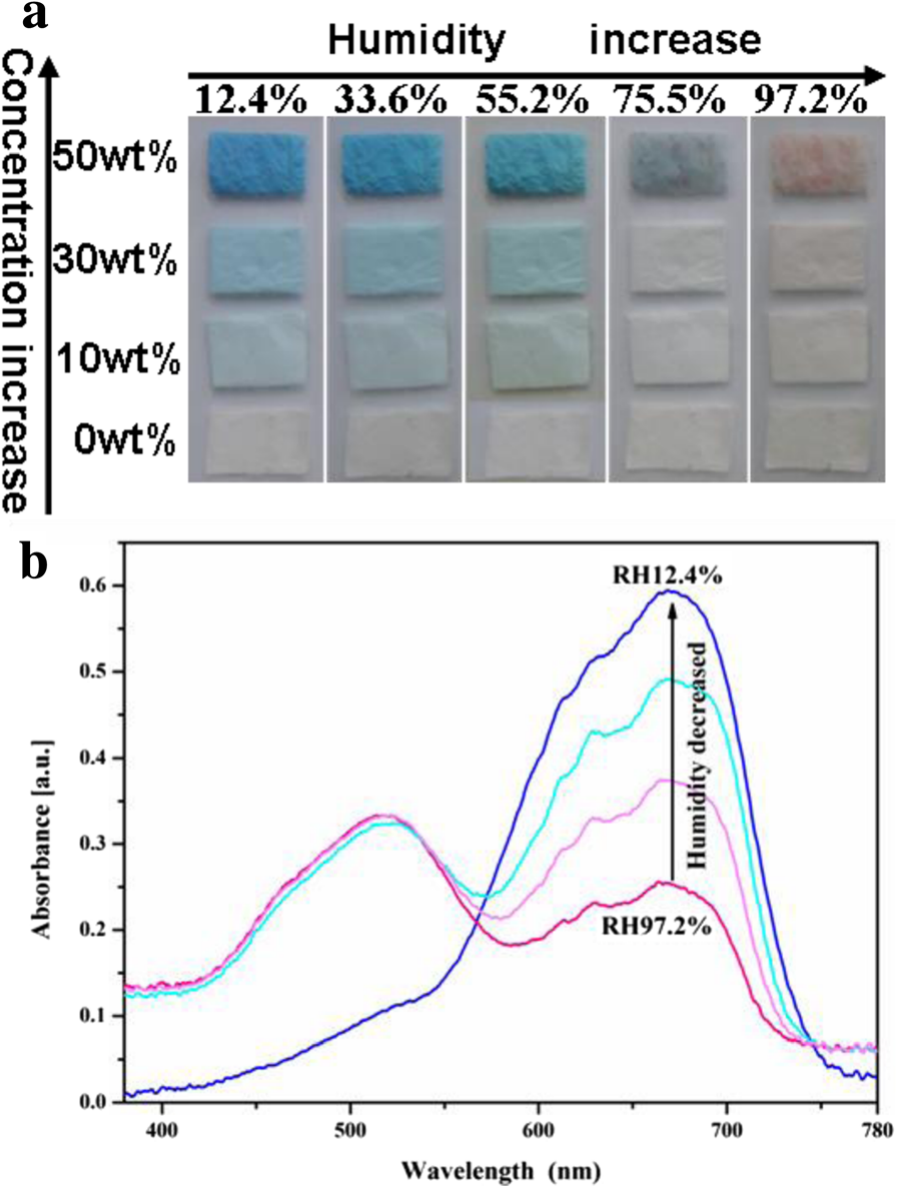
**a** The photographs of PA66/CoCl_2_·6H_2_O NFMs containing different concentrations of CoCl_2_·6H_2_O: 0, 10, 30, and 50 wt% in different RH. **b** Humidity dependence of visible absorption spectra (380–780 nm) of PA66/CoCl_2_·6H_2_O (50 wt%)

Figure 2a shows the color changes of the electrospun PA66/CoCl_2_ NFMs with different concentrations of CoCl_2_·6H_2_O NFMs exposure to different RH. As can be seen that the color of pure PA66 NFM is white, while the colors of PA66/CoCl_2_ NFMs in low humidity are blue, and as the concentration of cobalt chloride changed from 10 to 50 wt%, the color of the PA66/CoCl_2_ NFM became more and more concentrated. As humidity increases, especially for 50 wt% CoCl_2_·6H_2_O NFM, the blue color of the NFM gradually fades and turns to pink in the 97.2% RH.

In order to ensure the colors of PA66/cobalt chloride NFM exposed to different relative humidity conditions, visible absorption spectra (380–780 nm) of the 50 wt% CoCl_2_·6H_2_O-doped NFM following exposure to variable RH were examined as displayed in Fig. 2b. It was found that the absorbance of the hybrid NFM lying in the range of 410–550 nm corresponds to the spectral characteristics of CoCl_2_·6H_2_O [7]. Similarly, there is a weak absorption bands located at 580–750 nm (yellow and red light) with absorbance peak at ~670 nm, which may be caused by a small amount of residual anhydrous cobalt chloride inside the fibers. As the humidity decreases, the absorption bands of 580–750 nm are gradually sharpening, which correspond to the pink faded and turn to blue by degrees. Until 12.4% RH, the absorbing peak from 410 to 550 nm of the NFM disappears, and the absorption peak of 710 nm reaches a maximum value, which corresponds to the spectral characteristics of anhydrous CoCl_2_ [7]. It suggests that in different RH, the PA66/CoCl_2_·6H_2_O NFM shows different colors, indicating the composite NFM have potential application in humidity visualization hygrometer.

### Humidity Sensing Characteristics by QCM

Sensors based on QCM technique has been attracted a lot of interests due to its advantages of high sensitivity and reliability [14, 23, 24]. The operating principle of these sensors is primarily associated with the adsorption of the water molecules on the surface of the QCM electrode, inducing variation in the molar response of a quartz crystal, which lead to the resonance frequency shift [14, 25]. Consequently, we also selected QCM for further humidity sensitivity measurement.

### Sensitivity

**Fig. 3:**
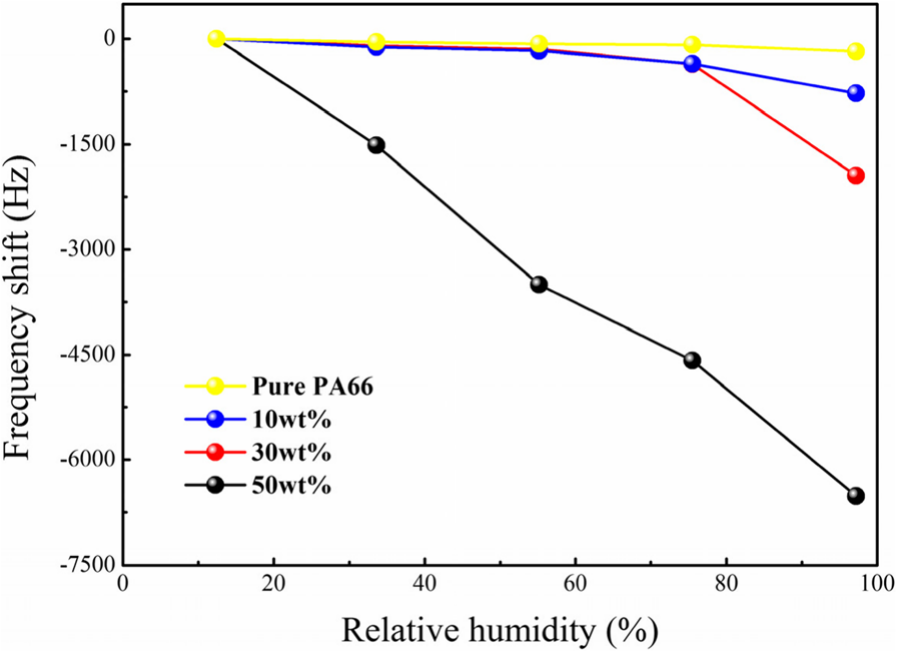
The relationship between the frequency shift and the corresponding relative humidity at different concentration of CoCl_2_·6H_2_O on PA66 NFMs measured by humidity ascending mode

Figure 3 shows the QCM frequency response curves of PA66/CoCl_2_ NFMs with different concentrations of CoCl_2_ under different RH. As can be found that the resonant frequency of the pure PA66 NFM did not show obvious change even at 97.2% RH, revealing that pure PA66 NFM was weakly sensitive to humidity. In contrast, the resonant frequency of the PA66 mixed with cobalt chloride NFMs sensors obviously shifted. The resonant frequencies of all the three sensors were negative growth corresponded to the increasing of humidity. The frequency shifts of the PA66/CoCl_2_·6H_2_O (10 wt%) NFM sensor and the PA66/CoCl_2_·6H_2_O (30 wt%) NFM sensor are very close and increased slowly below 75.5% RH. After exceeded 75.5% RH, the frequency shifts of the 10 and 30 wt% NFM sensors increased in a steep manner, which maybe due to that the complexes (Co) shielded by the PA66 molecular chain in low-RH regions and liberated in the high hydrated state in the high RH region [26]. And at 97.2% RH, the maximum frequency shifts were −777 and −1944 Hz for 10 and 30 wt% NFM sensors, respectively. As the concentration of CoCl_2_·6H_2_O further increased, the shielding effect was weakened; consequently, the resonant frequency shift of the 50 wt% NFM sensor was linear negative increase with the increase of RH and its regression coefficient *R* ^2^ reached 0.9937. Its maximum frequency shift was −6519.5 Hz at 97.2% RH. The results indicating that incorporation of cobalt chloride can increase the humidity sensitive of the QCM-based NFMs sensors and the sensing response increased with increasing concentration of cobalt chloride in the NFMs, and there were more absorption sites [10, 27] on the NFM due to the higher concentration of CoCl_2_ which increased the total amount of absorption water molecules. Based on this discovery, the future investigations about coating CoCl2 on PA66 nanofibers using the modified coaxial and triaxial electrospinning processes are worthy of conducting [28–31].

### Repeatability and Humidity Hysteresis Characteristic

**Fig. 4:**
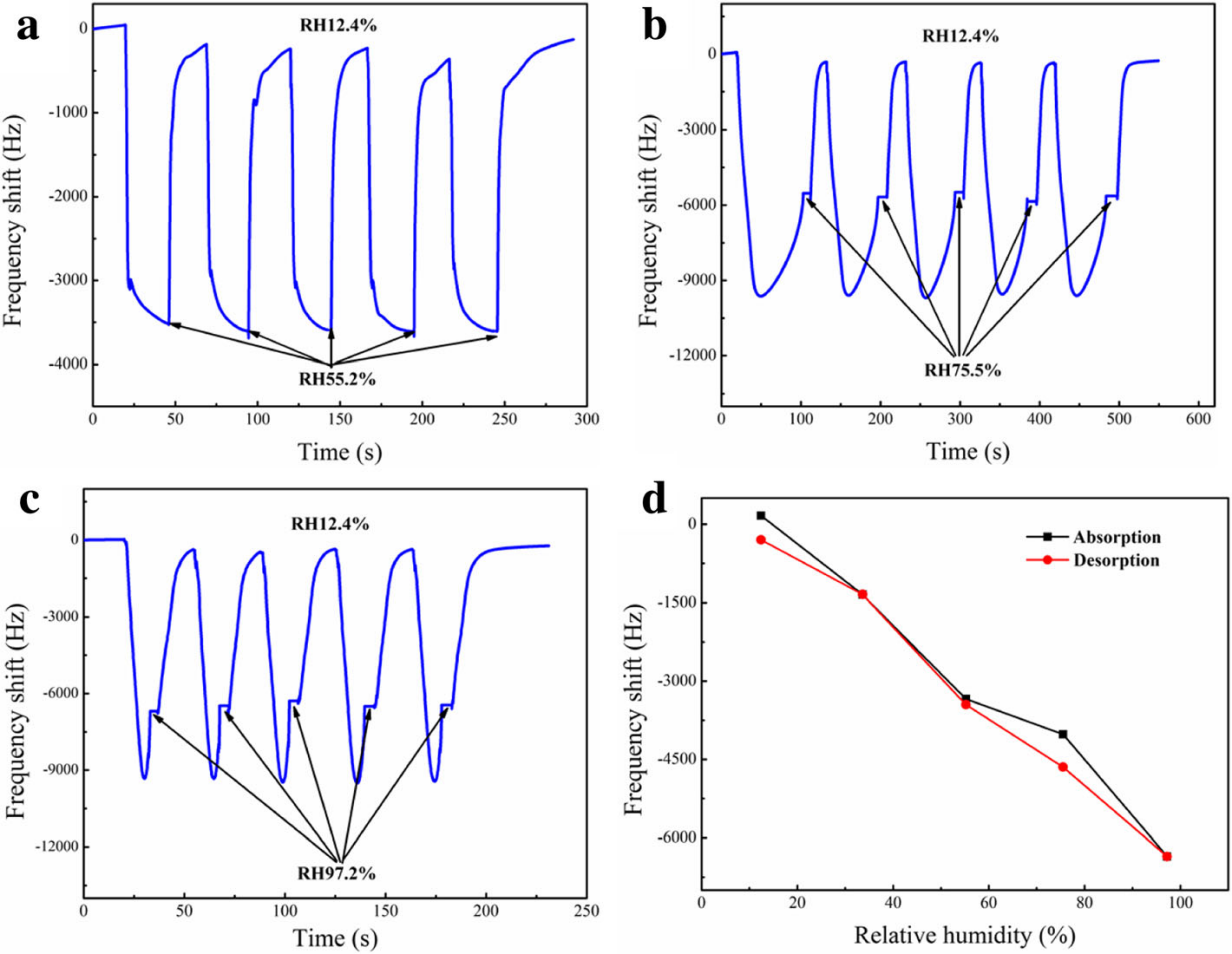
The repeatability characteristic curves of QCM-based PA66/CoCl_2_·6H_2_O (50 wt%) NFM sensor for 5 cycles of RH adsorption–desorption **a** between 12.4 and 55.2% RH, **b** between 12.4 and 75.5% RH, and **c** between 12.4 and 97.2% RH. **d** Humidity hysteresis characteristic of QCM-based PA66/CoCl_2_·6H_2_O (50 wt%) NFM sensor

It is well known that response and recovery behavior is an important characteristic for evaluating the performance of humidity sensors which corresponding to the water molecule adsorption and desorption processes [32]. Moreover, sensor repeatability refers to the successive runs using a single sensor to evaluate discrepancies in its response [33]. To investigate this performance, the PA66/CoCl_2_·6H_2_O NFM humidity sensors were alternately placed into different humidity bottles. The response and recovery characteristic curves for one cycle from three humidity differences: 12.4–55.2% RH, 12.4–75.5% RH, and 12.4–97.2% RH, with the PA66/CoCl_2_·6H_2_O (10, 30, and 50 wt%) NFMs QCM sensors were shown in Additional file 1: Figure S1 and Table S1. To investigate the sensors’ repeatability, the sensors were tested in two fixed humidity levels repeatedly. In this test, the PA66/CoCl_2_·6H_2_O (50 wt%) NFM sensors’ repeatability was tested in three humidity differences which corresponded to 12.4–55.2% RH, 12.4–75.5% RH, and 12.4–97.2% RH for five cycles showed as a representative (Fig. 4a–c). In these three conditions, whenever the sensor was switched to a humidity condition, the frequency promptly changed and rapidly reached a relative stable value, which was very close to the original value. Each cycle had a similar curved shape, and each stable value was very close. It suggests that the PA66/CoCl_2_·6H_2_O NFM sensors have good reproducibility in different humidity differences for humidity sensing.

Sensor hysteresis is examined to obtain some information about the reliability [14, 34]. Figure 4d shows the PA66/CoCl_2_·6H_2_O (50 wt%) NFM sensors’ humidity hysteresis characteristic. The black line in the figure was measured from low RH to high RH (from 12.4 to 97.2%), i.e., for the adsorption process, and the red line represents desorption process (measured in the opposite direction). The curves between adsorption process and desorption process have good coincidence for the as-spun sensor. The maximum humidity hysteresis was 9.9% (at about 75.5% RH) for PA66/CoCl_2_·6H_2_O NFM sensor with concentration of 50 wt% CoCl_2_·6H_2_O, indicating a good reliability of these humidity sensors, and the other humidity hysteresis curves of electrospun PA66/CoCl_2_·6H_2_O NFM with different CoCl_2_·6H_2_O concentration were shown in Additional file 1: Figure S2. Moreover, the stability of the sensor was also examined showing in Additional file 1: Figure S3.

### Morphology of the As-Spun Membranes

**Fig. 5:**
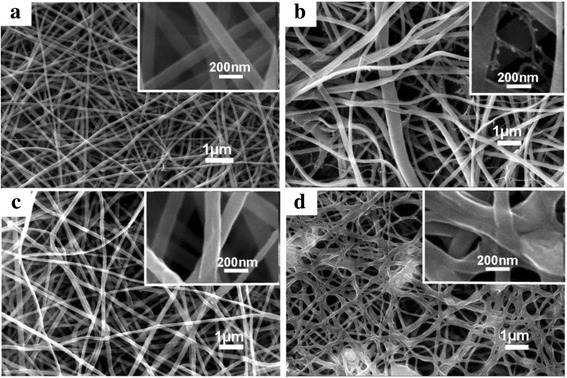
SEM images of **a** pure PA66 NFM, **b** PA66/CoCl_2_·6H_2_O (10 wt%) NFM, **c** PA66/CoCl_2_·6H_2_O (30 wt%) NFM, and **d** PA66/CoCl_2_·6H_2_O (50 wt%) NFM

As mentioned in the background, the high humidity sensitivity of electrospun PA66/CoCl_2_·6H_2_O NFM may due to their microstructures. Consequently, the SEM and TEM images of the as-spun NFMs samples are examined. As displayed in Fig. 5, these SEM images indicate that all of these NFMs with different concentrations of CoCl_2_·6H_2_O were the form of nonwoven mats with randomly oriented fibers. Figure 5a shows PA66 fibers have a relatively uniform diameter of about 100 nm. As showed in Fig. 5b, the diameter distribution of PA66/CoCl_2_·6H_2_O (10 wt%) fibers was about 100 to 500 nm. This might due to the increase of the solution conductivity leading to the increase of the solution-jet instability in the electric field [35]. In addition, in Fig. 5b, we found some smaller diameter (about 25 nm) nanofibers, namely, spider-net-like structure, which was shown in the inset of Fig. 5b more clearly. The formation of the spider-net-like structure may be due to the increased ionization of the polymer solution in the presence of cobalt ions (Co^2+^) and chloride ions (Cl^−^) during electrospinning process. Formic acid, a polar monoprotic solvent with high dielectric constant, is capable of attacking the lactam of nylon to produce a series of short chain oligomer/monomer ions (–CONH^2+^–) [36]. The Cl^−^ and Co^2+^ could further increase the concentration of ions in the electrospinning solution. The increased of ions could initiate the splitting up of subnanofibers from the main fibers and form the spider-net-like structure during electrospinning [37]. The PA66/CoCl_2_·6H_2_O (30 wt%) fibers were relatively uniform with a diameter of about 100 nm (Fig. 5c). Figure 5d shows the surface morphology of PA66/CoCl_2_·6H_2_O (50 wt%) NFM. The further increased in the CoCl_2_·6H_2_O (50 wt%) concentration led to a fiber-sticking structure which is more clearly showed in the inset of Fig. 5d. This fiber morphology could be caused by the localized charge effects on the surface of the fibers and slow evaporation of solvent from the fibers [38, 39].

**Fig. 6:**
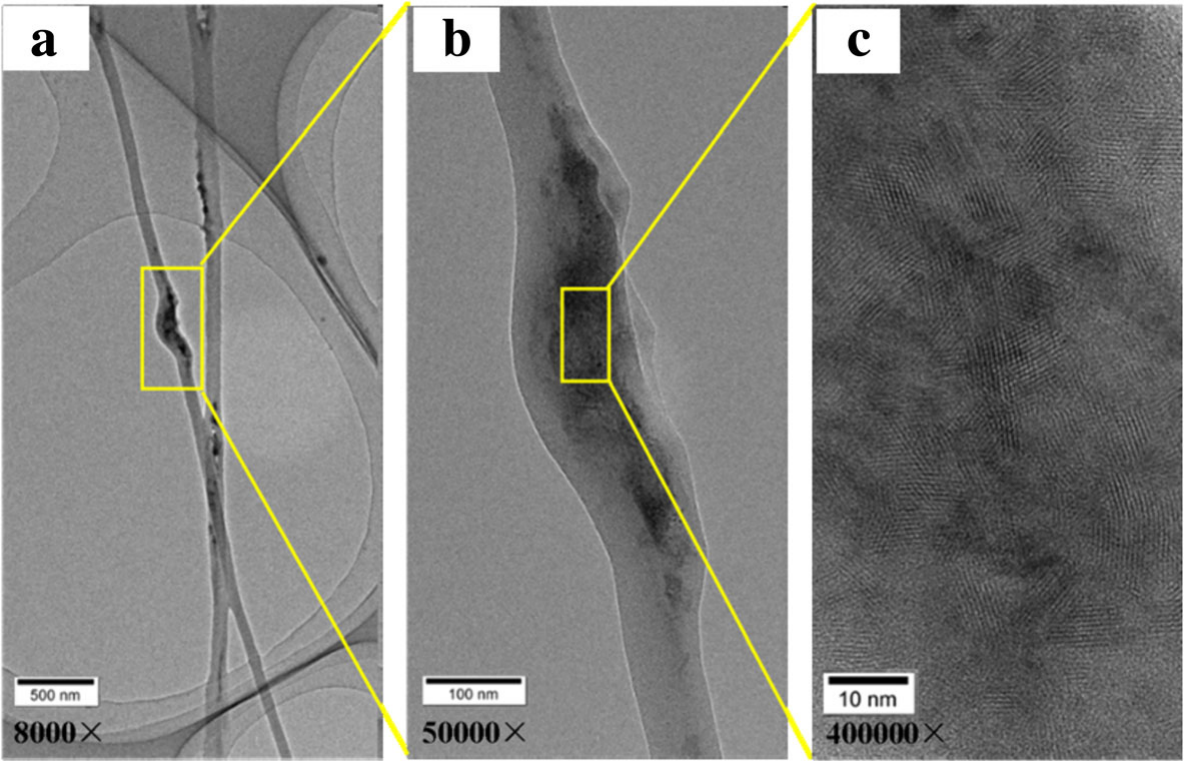
TEM images of PA66/CoCl_2_·6H_2_O (50 wt%) fibers: **a** magnified 8000 times and **b** magnified 50000 times. **c** HRTEM image of cobalt chloride particles

Figure 6 shows the TEM images of the PA66/CoCl_2_·6H_2_O (50 wt%) fibers. From Fig. 6a and its partial enlarged view Fig. 6b, we could see aggregated cobalt chloride particles dispersed in the PA66 nanofibers. Figure 6c is the high-resolution TEM (HRTEM) image of the cobalt chloride particles. From this image, a lot of lattice stripes with grain size of nearly 6 nm assigned to the crystalline cobalt chloride were observed. On the other hand, some regions were not lattice stripes, suggesting that partial cobalt chloride has an amorphous structure.

### FT-IR Spectra and Humidity Sensing Mechanism

**Fig. 7:**
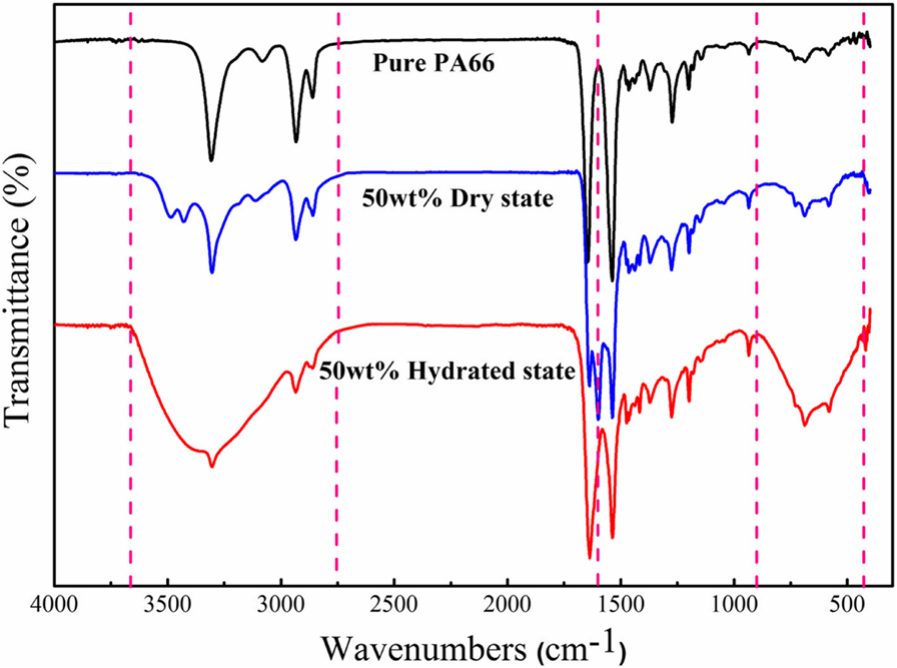
FT-IR spectra of pure PA66 NFM and PA66/CoCl_2_·6H_2_O (50 wt%) NFM in dry and hydrated states

Figure 7 shows the FT-IR spectra of the pure PA66 NFM and the PA66/CoCl_2_·6H_2_O (50 wt%) NFM in the dry and hydrated states. Comparing the spectra in dry and hydrated states, there are two obvious absorption bands located at 430–900 and 2750–3660 cm^−1^ in the dehydration curve. The absorption bands at 430–900 cm^−1^ are attributed to the bending and rocking modes of Co–OH_2_ (cobalt-water bond) and the rocking mode of O–H···Cl (hydrogen bonding with chlorine). The bands at 2750–3660 cm^−1^ correspond to the stretching mode of Co–OH_2_ [40, 41]. It indicates that the cobalt chloride in NFM lost crystalline water in low humidity and absorbed crystalline water in high humidity. In contrast, there is a pronounced peak at about 1600 cm^−1^ in the dry state, which cannot found in the hydrated state spectrum as well as the pure PA66 spectrum. It seems that the addition of CoCl_2_ in PA66 membrane could lead to the formation of coordination bonds between Co ions and PA66 molecular chain (probably the peptide groups), which caused the 1600 cm^−1^ peak in the dry PA66/CoCl_2_ NFM; as the NFM adsorbed water molecules, these coordination bonds are gradually destroyed so that this peak disappears in the hydrated NFM [26].

The humidity sensitive characteristics of the hybrid PA66/cobalt chloride NFMs mainly come from the cobalt chloride. When exposed to dry atmosphere, the cobalt chloride loses its crystalline water, conversely, in a wet environment it absorbs water molecules and turns into hydrated cobalt chloride. In this process, the color of PA66/cobalt chloride NFM changes. Moreover, in the process of dehydration and hygroscopic, the mass of the NFMs also changes, which leads to an according change of resonant frequency. In addition, it is noted that the PA66 has very small contribution to the mass change because the pure PA66 NMF can only cause a small frequency shift when RH changes.

## Conclusions

In summary, hybrid PA66/cobalt chloride humidity sensitive colorimetric nanofibrous membranes have been fabricated successfully by electrospinning. The influences of CoCl_2_ concentrations on morphology, color, and humidity sensitivity of the NFMs have also been systematically studied. The hybrid membranes exhibit different colors in different humidity conditions, indicating that PA66/cobalt chloride NFMs have promising application in visual hygrometer. Furthermore, PA66/cobalt chloride NFMs deposited on QCM also exhibit interesting humidity sensing properties. Since the nanofibrous structure may increase the sensing area and surface activity, the QCM-based humidity sensor shows high sensitivity, fast response, and recovery time, good reproducibility in moisture-sensitive and longtime stability in a stable humidity environment with a little frequency shift. These results indicate that the PA66/cobalt chloride NFM sensor has potential applications in humidity detection.

## Additional file

Additional file 1: Supporting information. (DOC 475 kb)
