# Supplementary material for: Colorimetric Humidity Sensors Based on Electrospun Polyamide/CoCl2 Nanofibrous Membranes
Source: Nanoscale Res Lett. 2017 May 19;12:360. doi: 10.1186/s11671-017-2139-0 (PMC5438331; doi:10.1186/s11671-017-2139-0)
Supplement: Additional file 1: — Supporting information. (DOC 475 kb) [file 11671_2017_2139_MOESM1_ESM.doc]

**Supporting Information**

**Colorimetric humidity sensors based on electrospun polyamide/CoCl_2_ nanofibrous membranes**

**S1. Response and recovery characteristics**

It is well known that response and recovery behavior is an important characteristic for evaluating the performance of humidity sensors which corresponding to the water molecule adsorption and desorption processes [1]. To investigate this performance, the PA66/CoCl_2_·6H_2_O NFM sensors were alternately placed into different humidity bottles. We have carried out humidity response and recovery experiments in three humidity differences: 12.4%-55.2% RH, 12.4%-75.5% RH, and 12.4-97.2% RH, respectively. Fig. S1 depicts the response and recovery characteristic curves for one cycle based on the PA66/CoCl_2_·6H_2_O (10 wt%, 30 wt% and 50 wt%) NFMs QCM sensors. All of these figures show that when changing RH from 12.4% to a higher value (55.2%, 75.5%, 97.2%) the sensor mixed more cobalt chloride in NFM exhibiting a bigger frequency variation width (Frequency shift: 10 wt% < 30 wt% < 50 wt%). It indicated that the moisture absorption capacity was higher with the increasing concentration of cobalt chloride in the NFM. Interestingly, in Figs. S1b and S1c, when moved the PA66/CoCl_2_·6H_2_O (50 wt%) NFM sensor from 12.4% RH to 75.5% RH or 97.2% RH, the sensor would absorb excessive moisture and then it quickly released this excessive absorption of moisture to reach equilibrium. This phenomenon is fully manifested by the curves within the yellow boxes (Fig. S1b-c). However, the phenomenon of excessive moisture absorption cannot be found in the curves of 10 wt% NFM sensor and 30 wt% NFM sensor. It suggested that the NFMs were more sensitive to moisture if there were higher concentrations of cobalt chloride in the NFMs. As seen in Fig. S1a, the phenomenon of excessive moisture absorption after moved the 50 wt% NFM sensor from 12.4% RH to 55.2% RH was not apparent. And the excessive absorption process of 12.4% to 97.2% RH was more intense than that of 12.4% to 75.5% RH because the former has a greater curvature curve (the curves within the yellow boxes). Maybe a greater humidity difference was one of the reasons for excessive moisture absorption.

The response time and recovery time of these three cases are showed in the radar chart (Fig. S1d) for comparison, and the specific values are listed in Table S1. These values were obtained by a sensor to achieve 90% of the total signal change in the case of adsorption and desorption,[2] except the cases of 12.4%-75.5% RH adsorption and 12.4%-97.2 RH adsorption because they had an oscillation curve (excessive moisture absorption curve). So, in these two cases we used the time to reach steady-state as the specific value. As can be seen from the figure, the desorption times were shorter than the adsorption times in all of these cases. The maximum adsorption time was 65.4 s (12.4%-75.5% RH, adsorption, 50 wt%, steady-state), the rest of the adsorption times were within 30 s. The minimum desorption time was 3.2 s (12%-97% RH, desorption, 30 wt%) and the maximum desorption time was 11 s (12%-97% RH, desorption, 50 wt%). Compared with others’ work, such as QCM-based polyethyleneimine/polyamide 6 (PEI-PA6) nano-fiber/net humidity sensors (the response and recover times are 120 s and 50 s, respectively, with RH changing from 2% to 35% ) [3], the response and recovery time of our QCM-based PA66/CoCl_2_·6H_2_O NFM sensors is much lower. It can be concluded that these PA66/CoCl_2_·6H_2_O NFM sensors had a excellent response and recovery performance.


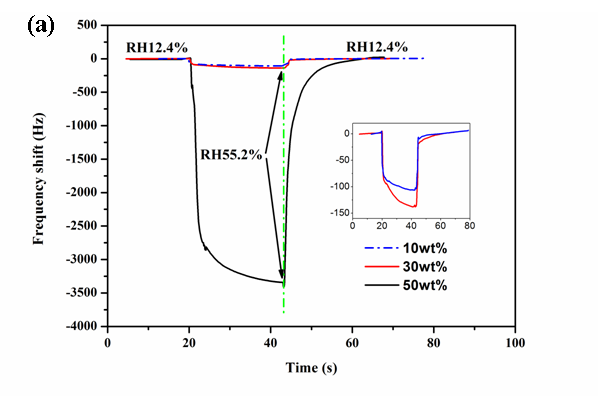


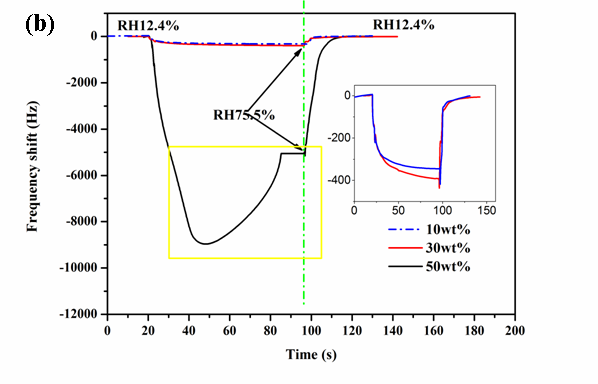


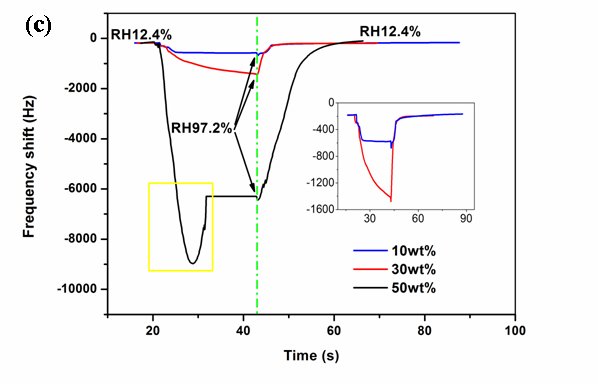


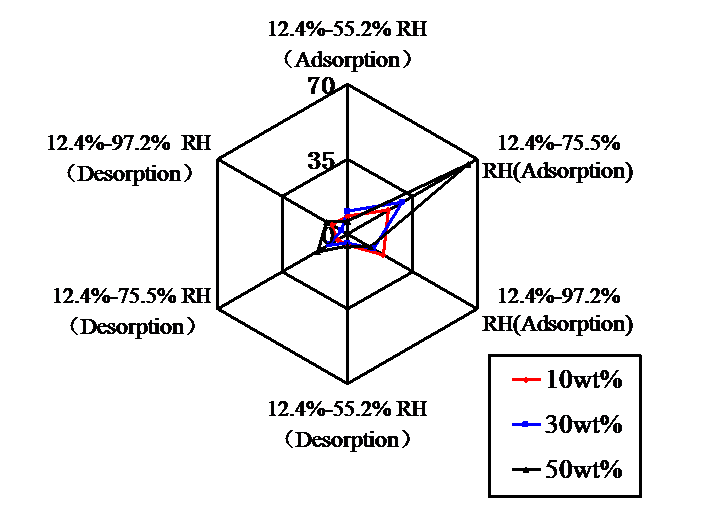


**(d)**

**Fig. S1** Response and recovery characteristic curves of different CoCl_2_·6H_2_O concentrations of hybrid PA66/CoCl_2_·6H_2_O NFM coated QCM sensors with RH changing (a) from 12.4% to 55.2%, (b) from 12.4% to 75.5% and (c) from 12.4% to 97.2%. (d) The radar chart of response and recovery times.

**Table S1** Response and recovery time of QCM-based PA66/CoCl_2_·6H_2_O (10 wt%, 30 wt% and 50 wt%) NFM sensors with RH changing of 12.4%-55.2% RH, 12.4%-75.5% RH and 12.4-97.2% RH. The red values represent the response time to reach steady-state.

**
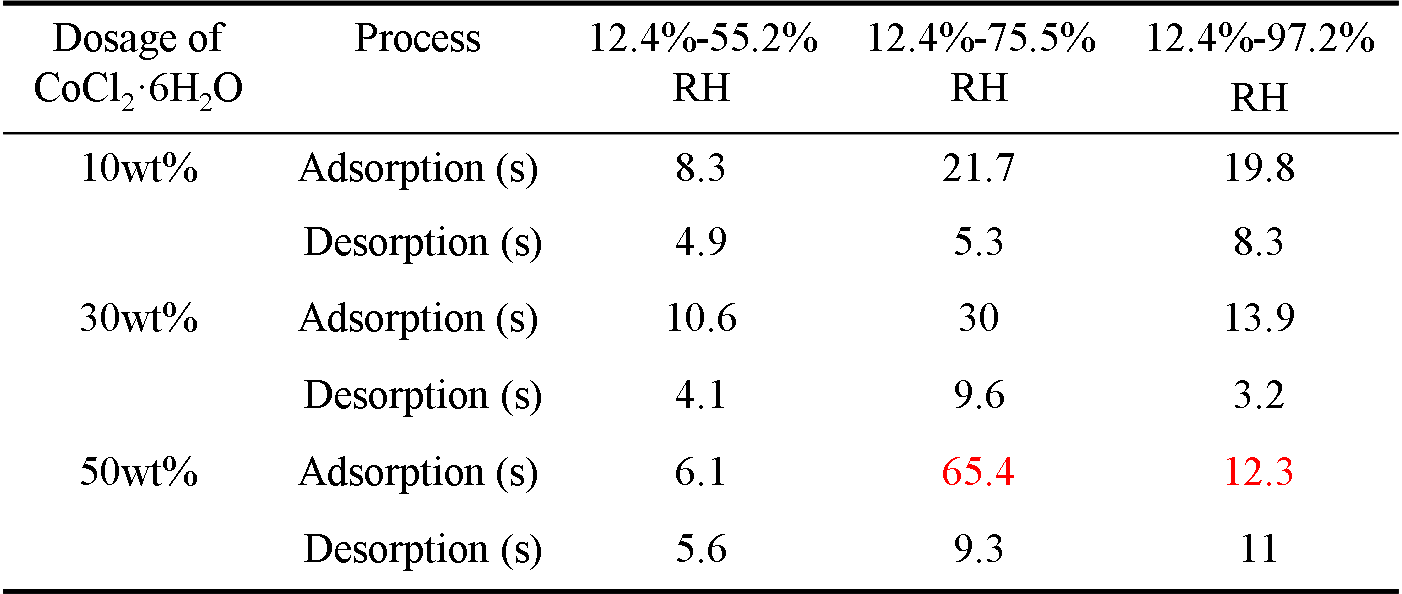
**

**S2 Humidity Hysteresis characteristic**

**
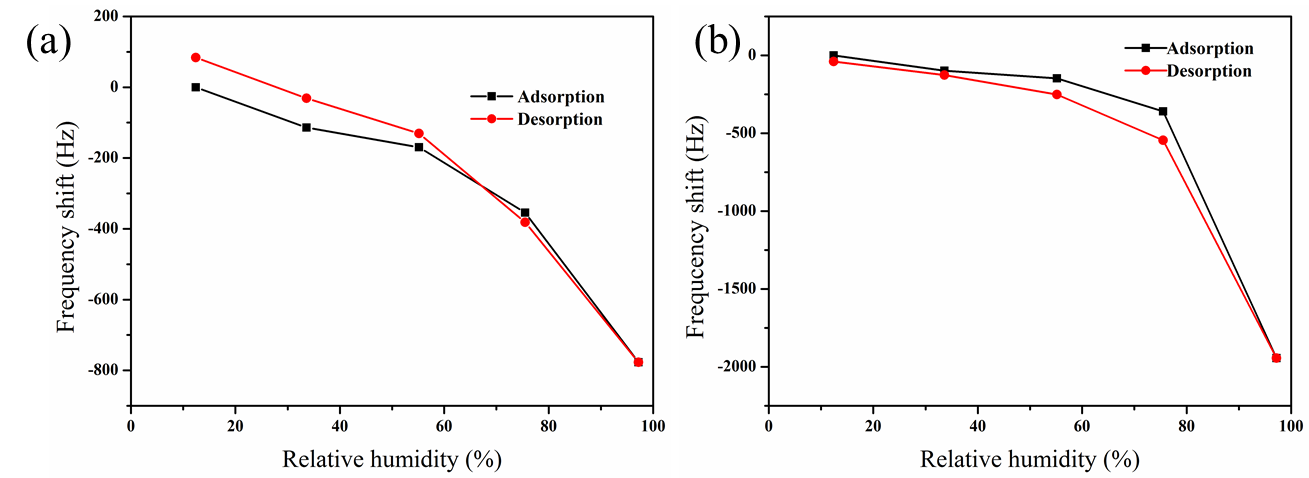
**

**Fig. S2** Humidity hysteresis characteristic of QCM-based (a) PA66/CoCl_2_·6H_2_O (10 wt%) NFM sensor, (b) PA66/CoCl_2_·6H_2_O (30 wt%) NFM sensor. The maximum humidity hysteresis were 10.8% (at about 12.4% RH) and 9.5% (at about 75.5% RH) for PA66/CoCl_2_·6H_2_O NFM sensor with concentration of 10 wt% and 30 wt% respectively.

**S3 Stability**

In order to test the stability of the sensor, we measured the frequency shift of the PA66/CoCl_2_·6H_2_O (50 wt%) NFM sensor working under a low humidity level (12.4% RH) and a high humidity level (75.5%RH) for a long time (120 min). In Fig. S3, the sensor’s frequency shifts were less than 7 Hz within 120 min and the variation of frequency shift with respect to the maximum frequency shift (-6519.5 Hz) was about 0.11%. It indicates that the QCM-based PA66/CoCl_2_·6H_2_O NFM sensor was very stable under a fixed humidity environment.

**
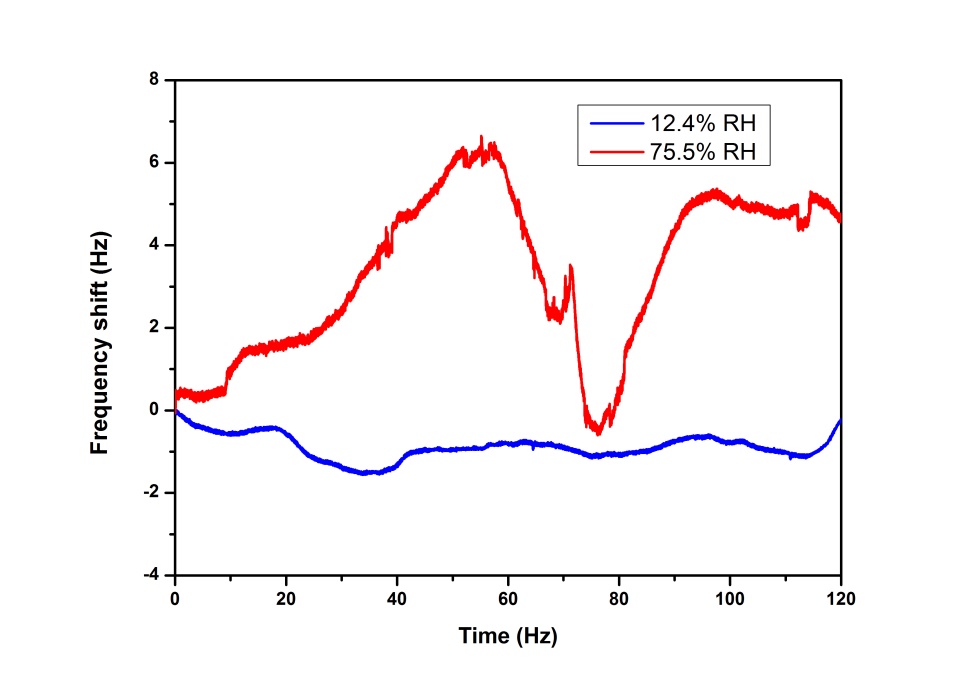
**

**Fig. S3** The frequency shift of the QCM-based PA66/CoCl_2_·6H_2_O (50 wt%) NFM sensor at 12.4% RH and 75.5% RH environment for 120 min.

**References**

[1] Qi Q, Zhang T, Yu Q J, Wang R, Zeng Y, Liu L, Yang H B, 2008 Sensors and Actuators B **133** 638-643.

# [2] S. Agarwal, G.L. Sharma, 2002 Sensors and Actuators B 85 205-211.

# [3] X.F. Wang, B. Ding, J.Y. Yu and M.R. Wang, 2011 J. Mater. Chem. 21 16231–16238.
